# *Tuberpulchrosporum* sp. nov., a black truffle of the Aestivum clade (Tuberaceae, Pezizales) from the Balkan peninsula

**DOI:** 10.3897/mycokeys.47.32085

**Published:** 2019-02-20

**Authors:** Elias Polemis, Georgios Konstantinidis, Vassiliki Fryssouli, Monica Slavova, Triantafyllos Tsampazis, Vasileios Nakkas, Boris Assyov, Vasileios Kaounas, Georgios I. Zervakis

**Affiliations:** 1 Agricultural University of Athens, Laboratory of General and Agricultural Microbiology, Iera Odos 75, 11855 Athens, Greece Agricultural University of Athens Athens Greece; 2 Agiou Kosma 25, 51100 Grevena, Greece Unaffiliated Grevena Greece; 3 4 Krivolak Street, Sofia, 1000, Bulgaria Unaffiliated Sofia Bulgaria; 4 Aristotelous 106, Maniaki, 52100 Kastoria, Greece Unaffiliated Kastoria Greece; 5 Konstantinou Davaki 218, Neochoropoulo, 45500 Ioannina, Greece Unaffiliated Ioannina Greece; 6 Institute of Biodiversity and Ecosystem Research, Bulgarian Academy of Sciences, 2 Gagarin Str, Sofia, 1113, Bulgaria Institute of Biodiversity and Ecosystem Research, Bulgarian Academy of Sciences Sofia Bulgaria; 7 Sokratous 40, 19016 Artemis, Attica, Greece Unaffiliated Artemis Greece

**Keywords:** Ascomycota, Tuberaceae, truffle, ectomycorrhizal fungi, taxonomy, phylogeny, fungal diversity

## Abstract

Knowledge on the diversity of hypogeous sequestrate ascomycetes is still limited in the Balkan Peninsula. A new species of truffle, *Tuberpulchrosporum*, is described from Greece and Bulgaria. Specimens were collected from habitats dominated by various oak species (i.e. *Quercusilex*, *Q.coccifera*, *Q.robur*) and other angiosperms. They are morphologically characterised by subglobose, ovoid to irregularly lobed, yellowish-brown to dark brown ascomata, usually with a shallow basal cavity and surface with fissures and small, dense, almost flat, trihedral to polyhedral warts. Ascospores are ellipsoid to subfusiform, uniquely ornamented, crested to incompletely reticulate and are produced in (1–)2–8-spored asci. Hair-like, hyaline to light yellow hyphae protrude from the peridium surface. According to the outcome of ITS rDNA sequence analysis, this species forms a distinct well-supported group in the Aestivum clade, with *T.panniferum* being the closest phylogenetic taxon.

## Introduction

The genus *Tuber* F.H. Wigg. (Ascomycota, Pezizales, Tuberaceae) is globally famous and historically appreciated for the production of hypogeous ascomata, known as ‘truffles’; several of them are highly prized due to their unique aroma and culinary value. Moreover, the genus is known for the symbiotic ectomycorrhizal associations that its members form with several gymnosperm and angiosperm forest-tree species as well as with orchids (Riousset et al. 2001; Selosse et al. 2004; Mello et al. 2006; Trappe et al. 2009). Furthermore, truffles are also important for serving as a primary or supplementary source of nutrition for soil micro-fauna and several mammals (Hanson et al. 2003; Trappe and Claridge 2010; Schickmann et al. 2012).

A continuous interest in the study of this particular group has resulted in several recent reports on new *Tuber* species from various parts of the world (e.g. Crous et al. 2017; Fan et al. 2015; Guevara-Guerrero et al. 2018; Piña Páez et al. 2018). It is estimated that their number ranges between 180 and 220 (Zambonelli et al. 2016) nested in 11 major phylogenetic clades (Bonito et al. 2013). In particular, the Aestivum clade is composed of species associated with a large spectrum of host plants and are reported to occur in the Old World, i.e. Europe, North Africa and/or Asia (Jeandroz et al. 2008; Bonito et al. 2013; Payen et al. 2014). Indicative examples are *T.aestivum* Vittad. (the type species of the genus), *T.panniferum* Tul. & Tul., *T.malenconii* Donadini, Riousset, G. Riousset & G. Chev. and *T.mesentericum* Vittad., as well as *T.sinoaestivum* Zhang & Liu recently described from China (Zambonelli et al. loc. sit.; Zhang and Chen 2012). The morphologically diverse and economically important species *T.magnatum* Picco also forms part of this clade (Bonito et al. 2010a; 2013).

Although *Tuber* diversity is well documented in Europe (Bonito at al. 2010a, Ceruti et al. 2003, Jeandroz et al. 2008), the south-eastern part of the continent and especially the Balkan Peninsula was until recently poorly investigated. Indicative of this fact is that, by the end of the last century, only three *Tuber* species had been recorded in Greece (Zervakis et al. 1999). However, during the last two decades, an ever increasing interest in the collection of truffles led to a remarkable increase in the number of pertinent records (e.g. Diamandis and Perlerou 2008; Konstantinidis 2009; Agnello and Kaounas 2011; Alvarado et al. 2012a,b; Gyosheva et al. 2012); thus, to date, 15 *Tuber* spp. are reported from Greece. Similarly, only two *Tuber* spp. had been recorded in Bulgaria by the end of the last century; however, this number is fast-growing during the last few years and 14 species are currently known to exist (Dimitrova and Gyosheva 2008; Gyosheva et al. 2012; Lacheva 2012; Nedelin et al. 2016; Assyov and Slavova 2018). Regarding adjacent countries, 12 truffle species were reported to occur in Serbia, including one recently described (Marjanović et al. 2010; Milenković et al. 2015), while six *Tuber* spp. were recorded in Montenegro, five in FYROM and four in Albania (Pacioni 1984; Marjanović et al. 2010).

In the frame of this work, several truffle specimens originating from north and central continental Greece and from Bulgaria were studied with respect to their morphology and phylogenetic relationships to other *Tuber* taxa and a new species is hereby proposed.

## Methods

### Sampling and Morphological characterisation

Specimens used for this study were collected during 2008–2017 from north and central Greece (Regions of Epirus, Thessaly, Eastern Macedonia and Thrace, Western Greece and Attica), as well as from Bulgaria (Regions of Eastern Stara Planina and Black Sea coast). Specimens are deposited in the fungaria of the Laboratory of General and Agricultural Microbiology (Agricultural University of Athens, ACAM), of the Institute of Biodiversity and Ecosystem Research (SOMF) and the authors’ personal collections. Macroscopic characters such as size, peridium surface texture, colour and odour were observed in fresh ascomata. Colour coding and terminology is derived from the “Flora of British Fungi – Colour Identification Chart” (Royal Botanic Garden Edinburgh 1969).

Microscopic characters were examined by hand-cut sections on fresh and dried material, using a Zeiss Axioimager A2 microscope under bright field and Differential Interference Contrast (DIC) and an AmScope T360B. Microphotographs were taken with the aid of a mounted digital camera (Axiocam). Microscopic observations were performed in water, 3% (w/v) potassium hydroxide (KOH) and Melzer’s reagent. To assess the ascospore size, a minimum of 30 mature ascospores from each type of asci (2 to 8-spored) were measured and dimensions are provided as (minimum) average ± standard deviation (maximum); quotient (Q), i.e. length divided by the width, was calculated for each ascospore and the median value (Qm) is given. For scanning electron microscopy (SEM), ascospores were scraped from the hymenial surface and mounted on aluminium foil, which was then fixed on a microscope holder and sputter-coated with gold. Observations were performed in JEOL JSM-5510.

### DNA sequencing and Phylogenetic analyses

Total genomic DNA was extracted from herbarium specimens using the Nucleospin Plant II DNA kit (Macherey and Nagel, Germany) following the manufacturer’s protocol with minor modifications. The internal transcribed spacer (ITS) region of nuclear ribosomal DNA (nrDNA) was amplified using the primer combination ITS1/ITS4 (White et al. 1990). Polymerase chain reactions (PCR) were performed in 50 μl containing 50 ng DNA template, 0.25 μM of each primer, 0.2 mM of each dNTP, 1× HiFi Buffer (Takara BIO INC., Japan) and 1 U HiFi Taq DNA polymerase (Takara BIO INC., Japan). Conditions for PCR amplification were as follows: 94 °C for 5 min, followed by 35 cycles of 94 °C for 30 sec, 50 °C for 30 sec and 72 °C for 1 min, with a final extension at 72 °C for 10 min. PCR products were purified using Invitrogen PureLink kit (Thermo Fisher Scientific, Korea) and were submitted for sequencing to CeMIA SA (Larissa, Greece). DNA sequences were then visualised, manually edited and assembled using UGENE (Okonechnikov et al. 2012). Validated sequences, generated in this study, were deposited in GenBank under the accession numbers MK113975 to MK113982 (Table 1). Moreover, the percent sequence identity was estimated by using ClustalOmega (Sievers and Higgins 2018) through the EMBL-EBI portal.

A total of 62 *Tuber*ITS rDNA sequences were used for phylogenetic analysis by including eight sequences of *T.pulchrosporum* sp. nov. and 54 sequences from GenBank (nine of them representing type specimens) which correspond to 31 *Tuber* taxa mainly of European distribution (Table 1). *Choiromycesalveolatus* (Harkn.) Trappe (AF501258, EU697268) was used as the outgroup. Sequence alignment was performed through the online version of the multiple sequence alignment programme MAFFT v7 (Katoh and Standley 2013) by applying the Q-INS-I strategy and alignments were inspected and manually adjusted at misaligned sites by using MEGAX (Kumar et al. 2018). The pertinent matrix was deposited in TreeBASE under the accession number 23587.

**Table 1.** Details of ITS sequences deriving from *Tuberpulchrosporum* sp. nov. and from reference material used for the construction of the phylogenetic tree. Clades names are placed in the order they appear in Fig. 5.

Species/ Clade	Collection code	GenBank Accession No.	Origin	Reference
Excavatum Clade				
* Tuber fulgens *	M2435	HM485358	Italy	Bonito et al. 2010a
	HMT37	HM151976^*^	Austria	Urban et al. 2010
* Tuber excavatum *	SA1TE	KJ524533^*^	Poland	Hilszczanska et al. 2014
	JST62014	KX354295	Germany	Schiebold et al. 2017
Gennadii Clade				
* Tuber lacunosum *	AH39255	JN392212	Spain	Alvarado et al. 2012a
	AH38932	JN392213	Spain	Alvarado et al. 2012a
* Tuber gennadii *	B M1904	HM485361	Italy	Bonito et al. 2010a
	AH39251	JN392211	Spain	Alvarado et al. 2012a
	AH31113	JN392203	Spain	Alvarado et al. 2012a
	AH38957	JN392204	Spain	Alvarado et al. 2012a
Regianum Clade				
* Tuber bernardinii *	2172	KY420104	Italy	Merenyi et al. 2017
	NA	KY420105	Italy	Merenyi et al. 2017
* Tuber magentipunctatum *	MO793	KY420089	Italy	Merenyi et al. 2017
	ZB4293	JQ288909^**^	Hungary	Merenyi et al. 2017
* Tuber regianum *	ZB3081	KY420098	Slovakia	Merenyi et al. 2017
	erd-2590	KY420102	Spain	Merenyi et al. 2017
Macrosporum Clade				
* Tuber macrosporum *	Macro1	AF106885^*^	Italy	Rubini et al. 1998
	HMSFI_TUBMAC/141207A	FM205634^*^	Slovenia	Grebenc et al. 2008
Aestivum Clade				
* Tuber magnatum *	JT19460	HM485374	Italy	Bonito et al. 2010a
	GB12	JQ925645	Italy	Bonito et al. 2013
* Tuber malenconii *	MA:Fungi:28384/ 02MLC	FM205597^*^	Spain	Grebenc et al. 2008
	17110	JF908743	Italy	Osmundson et al. 2013
* Tuber sinoaestivum *	L4213	KY081688^*^		Wang and Wang 2016
	JP-Zhang-140	JN896355	China	Zhang et al. 2012
* Tuber aestivum *	TaeW016I-E134	AJ888090	Italy	Weden 2005
	S19	HQ706002	Slovakia	Gryndler et al. 2011
* Tuber uncinatum *	MA: Fungi: 24605	FM205618^*^	Spain	Grebenc et al. 2008
	228	AJ492199	Italy	Mello et al. 2002
* Tuber mesentericum *	CW105	HM485375	Sweden	Bonito et al. 2010a
	UASWS1612	KY197989^*^	Switzerland	Cochard et al. 2016
* Tuber panniferum *	–	AF132507		Roux et al. 1999
	JT12835	HM485380	Spain	Bonito et al. 2010a
*Tuberpulchrosporum* sp. nov.	1945 F8517	MK113981	Bulgaria	This work
	1961 F0388	MK113982	Bulgaria	This work
	VN091 (holotype)	MK113975	Greece	This work
	GK3801	MK113979	Greece	This work
	LT1183	MK113976	Greece	This work
	GK9408	MK113977	Greece	This work
	VK4482	MK113980	Greece	This work
	GK6538	MK113978	Greece	This work
Multimaculatum Clade				
* Tuber multimaculatum *	OSC 62169	HM485377	Spain	Bonito et al. 2010a
Rufum Clade				
* Tuber rufum *	1785	EF362475	Italy	Iotti et al. 2007
	S90	JF926123	Germany	Stobbe et al. 2012
Melanosporum Clade				
* Tuber pseudoexcavatum *	T14_HKAS44325b	GU979039	China	Chen et al. 2011
	Tpse-yn05	DQ329374	China	Wang et al. 2006
* Tuber regimontanum *	ITCV 909	EU375838	Mexico	Guevara et al. 2008
* Tuber indicum *	Ascocarpe I1	AF300822	China	Mabru et al. 2001
	HKAS 39501	AY514305	China	Zhang et al. 2005
* Tuber melanosporum *	SB2-6	MF693845	France	Schneider-Maunoury et al. 2018
	P_Qr	KP972070	Canada	Berch and Bonito 2016
Tumericum Clade				
* Tuber turmericum *	BJTC FAN475	KT758839	China	Fan et al. 2015
	BJTC FAN473	KT758837	China	Fan et al. 2015
Gibbosum Clade				
* Tuber oregonense *	DUKE GB284	FJ809874	USA	Bonito et al. 2010b
* Tuber gibbosum *	OSC 40964	FJ809863	USA	Bonito et al. 2010b
Maculatum Clade				
* Tuber maculatum *	A15	AM406673	Italy	El Karkouri et al. 2007
	Db-A	MH040280^*^		Sikora 2018
Latisporum Clade				
* Tuber latisporum *	HKAS 44315	DQ898183	China	Chen and Liu 2007
* Tuber pseudosphaerosporum *	BJTC Fan250	KF744063	China	Fan and Yue 2013
Puberulum Clade				
* Tuber cistophilum *	AH 39275	JN392231	Spain	Alvarado et al. 2012a
* Tuber borchii *	Tar042	KT165326	Italy	Belfiori et al. 2016
* Tuber sphaerospermum *	AH38930	JN392244	Morocco	Alvarado et al. 2012a
	AH39190	JN392246	Spain	Alvarado et al. 2012a
Outgroup				
* Choiromyces alveolatus *	22830	AF501258		Ferdman et al. 2005
	p612i	EU697268^*^		Gordon 2008

^*^ unpublished sequence.

^**^ this sequence appears as “T. regianum” in GenBank (unpublished; Merenyi et al. 2011).

Phylogenetic relationships of taxa were inferred by using maximum likelihood (ML) and Bayesian Inference (BI) through the CIPRES portal (www.phylo.org; Miller et al. 2010). ML analysis of the ITS dataset was conducted by RAxML v8.2 (Stamatakis 2014) with 1,000 bootstrap replicates and search for the best-scoring ML tree. BI analysis was performed by MrBayes v3.2.1 (Ronquist et al. 2012) and the General Time Reversible + Gamma (GTR+G) model was selected as the best model under the Akaike Information Criterion (AIC) implemented in MrModeltest v2.3 (Nylander 2004). To estimate posterior probabilities, 20,000,000 Markov chain Monte Carlo (MCMC) simulation generations were run in two parallel independent runs of four chains, one cold and three heated, with trees sampled every 1,000 generations and the first 25% of trees were omitted as burn-in. A 50% majority rule consensus tree was built and visualised with iTOL (Letunic and Bork 2016). Clades with bootstrap support (BS) ≥ 70% and Bayesian posterior probability (PP) ≥ 95% were considered as significantly supported.

## Results

### Taxonomy

#### Tuber pulchrosporum

Taxon classificationFungiORDOFAMILIA

Konstantinidis, Tsampazis, Slavova, Nakkas, Polemis, Fryssouli & Zervakis sp. nov.

MycoBank: MB 828883

GenBank: MK113975

Fig. 1a

##### Type.

GREECE. Ioannina Prefecture: Ioannina city, 39°36'39"N, 20°50'05"E, 500 m alt., in soil under a pure stand of *Quercuscoccifera* L., 27 Apr 2016, coll. V. Nakkas, VN091, holotype: ACAM 2016-007 (ACAM!); isotype: SOMF 29980 (SOMF!).

##### Diagnosis.

Ascomata 0.6–7(–10) cm in diam., subglobose, ovoid to irregularly lobed, usually with shallow basal cavity, surface with fissures and small, dense, almost flat trihedral to polyhedral warts, yellowish-brown to dark brown. Ascospores 25.0–37.0 × 18.2–25.6 μm in (1–)2–8-spored asci, ellipsoid to subfusiform on average, Qm=1.4, crested to incompletely reticulate. Hair-like, hyaline to light yellow-brown hyphae protruding from peridium surface.

*T.panniferum*, the closest phylogenetically-related species, produces smaller ascospores (23–26 × 18–20 μm), broadly ellipsoid to subglobose on average, with isolated warts; moreover, the peridium surface is woolly-felted due to the presence of dense rusty brown hair-like hyphae.

##### Etymology.

“*pulchrosporum*” refers to the uniquely distinct/impressive ornamentation of the ascospores.

##### Description.

**Figure 1.**
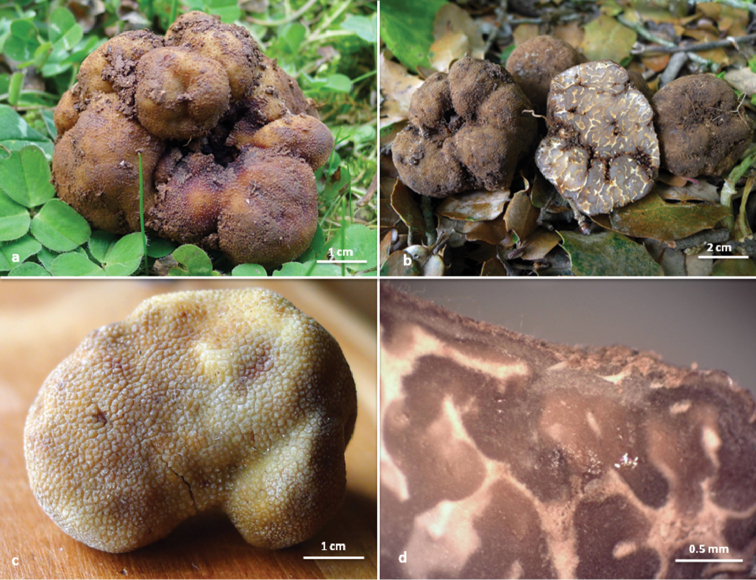
*T.pulchrosporum* sp. nov.: **a** ascomata in situ (holotype) **b** ascomata in situ (paratype) **c** detail of peridium surface (paratype) **d** section of peridium (paratype).

***Ascomata*** 0.6–7(–10) cm in diameter, tuberous, subglobose, ovoid to irregularly lobed, usually depressed with a shallow - occasionally prominent - basal cavity (excavated), covered up with whitish to yellowish rhizomorphs, fragile, initially greyish to yellowish-brown [fawn (29), sienna (11), fulvous (12)], darkening in maturity to brown [snuff brown (17), umber (18), bay (19), to date brown (24)] or with some shades of purple tinges [purplish date (22), purplish chestnut (21) to brown vinaceous (25)], sometimes with darker black [fuscous black (38)] spots, surface rarely almost smooth, usually rough, with fissures and small, dense, almost flat trihedral to polyhedral warts. *Gleba* with one of more cavities, initially pinkish-grey [vinaceous buff (31), clay pink (30)], then greyish-brown [milky coffee (28)], yellowish-brown [fulvous (12)], brown [snuff brown (17), umber (18), bay (19)], to purplish-brown in maturity [purplish date (22) to purplish chestnut (21)], with bay (19) to rusty tawny (14) coloured areas close to the cavity, marbled with relatively few and thick white veins, that sometimes are reddening (Fig. 1). *Odour* pleasant truffle-like.

**Figure 2.**
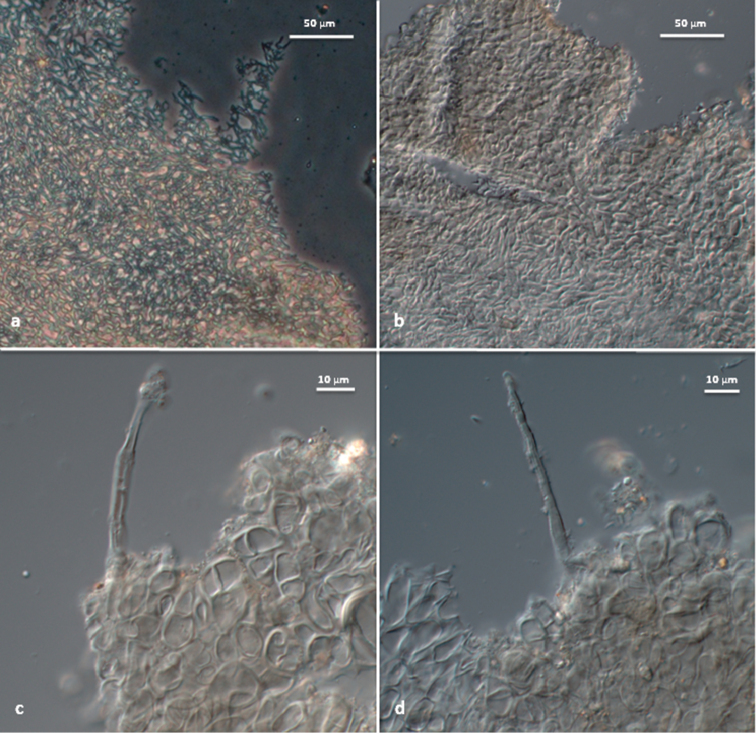
*T.pulchrosporum* sp. nov.: **a, b** peridium structure **c, d** hair-like hyphae on peridium surface.

***Peridium*** 120–370 μm thick, consisting of two layers; the outer layer 50–160 μm thick, pseudoparenchymatous, composed of yellowish-brown and subglobose inwards to subangular dark brown cells outwards; 4.0–16.3 × 2.5–13.2 μm, thick-walled (1.5–2.5 μm); the inner layer 70–210 µm, composed of pale yellow or hyaline and thick-walled, interwoven hyphae, 2–10 μm in diameter, forming an intricate texture, becoming agglutinated when dried. Surface with abundant isolated, hyaline to golden-yellow (in water or KOH), thick-walled hair-like hyphae (walls 1.0–1.5 μm), 30–140 μm long (occasionally exceeding 300 μm in Bulgarian specimens) and 2.5–4.5 μm broad at base, 1–2 septate (Figs 1, 2).

**Figure 3.**
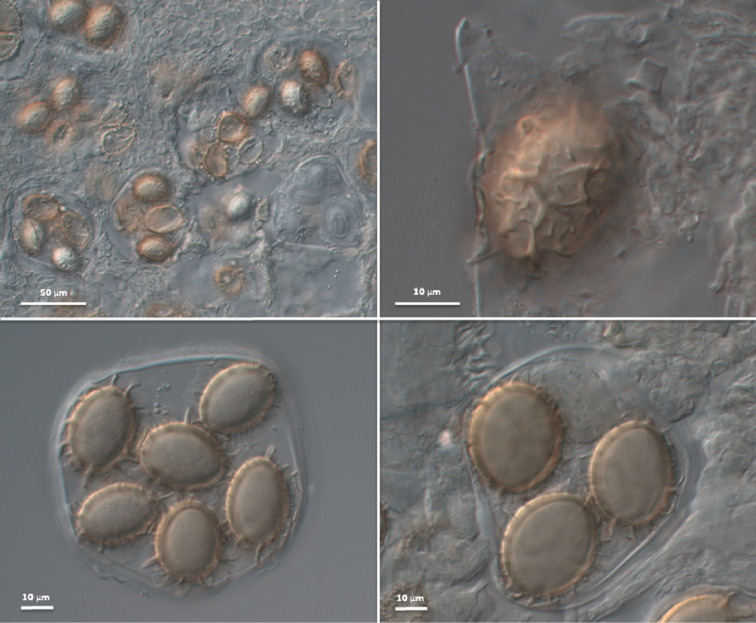
*T.pulchrosporum* sp. nov.: asci and ascospores.

**Figure 4.**
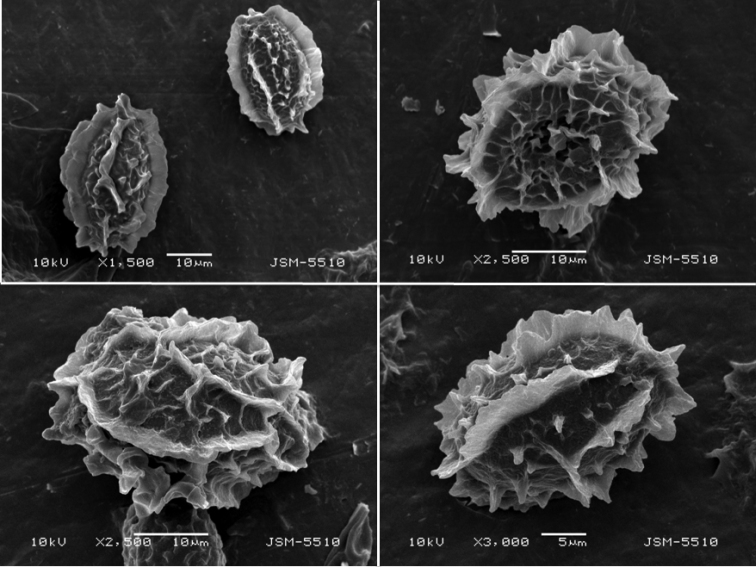
*T.pulchrosporum* sp. nov.: SEM of ascospores.

***Ascospores*** hyaline when young then yellowish, yellow-brown to brown, at most ellipsoid to subfusiform, some broadly ellipsoid, subglobose to globose, rarely almost limoniform in initial stages, thin-walled and smooth when young, becoming thick-walled at maturity, walls 2–3.5(–4) μm thick, usually crested to incompletely reticulate, measured (excluding the ornamentation) in the rare 1-spored asci (28–) 46.7±7.4 (–57) × (20–) 29.4±4.6 (–34) μm, in 2-spored asci (27–) 39.5±5.8 (–53) × (21–) 27.3±4.2 (–41) μm, in 3-spored asci (24–) 34.5±5.3 (–49) × (19–) 24.5±2.6 (–31) μm, in 4-spored (21–) 30.9±4.9 (–39) × (18–) 22.2±2.7 (–30) μm, in 5-spored asci (22–) 30.3±3.7 (–44) × (16–) 21.2±2.2 (–28) μm, in 6-spored asci (22–) 28.9±4.6 (–37) × (17–) 20.6±2.0 (–28) μm, in 7-spored asci (21–) 27.8±3.3 (–35) × (13–) 19.9±2.7 (–27) μm and in 8-spored asci (20–) 25.4±2.6 (–31) × (14–) 18.4±3.1 (–26) μm (Fig. 3); Q=1.0–2.2, Qm=1.43±0.19; ornamentation with (0–)1–2(–4) thick veins across the long axis with few to several transverse outgrowths, rarely almost completely reticulate in maturity and then with (0–)2–10(–15) meshes in the longitudinal direction; circumferentially with 22–42 conical warts, with pointed or blunt, straight or curved apices, rarely forked, 1.5–6(–8) μm tall (Fig. 4); not reacting with Melzer’s reagent. *Asci* (64–) 78–96 (–121) × (50–) 65–84 (–98) μm (excluding stalk), globose, subglobose, ellipsoid, rarely subangular, with a short stalk, 6.5–9(–15) × 6.5–7.5(–10.5) μm, (1–)2–8-spored (Fig. 3).

##### Distribution and ecology.

Hypogeous, in soil, appearing solitary or in small groups from March to June, under *Quercus* sp., *Q.coccifera* or *Q.ilex* L. or under *Carpinus* sp. or in mixed stands of *Quercus* sp. and *Pinusnigra* J.F. Arnold or of *Q.ilex* and *Pinushalepensis* Miller or of *Quercusrobur* L., *Corylus* sp., *Carpinus* sp. and *Acer* sp. It seems to be rather common in continental (northern and central) Greece, while it also occurs in the regions of Eastern Stara Planina and the Black Sea coast of Bulgaria.

##### Additional collections examined (paratypes).

GREECE. Xanthi Prefecture: Toxotes, in soil under a mixed stand dominated by *Q.coccifera*, 20 June 2008, GK3186b (ACAM 2010-127), coll. P. Panagiotidis. Aitoloakarnania Prefecture: Xiromero, in soil under pure forest of *Quercus* sp., 10 May 2009, GK3801 (ACAM 2010-129), coll. Ch. Chrysopoulos and K. Giatra (GenBank: MK113979); Xiromero, in soil under pure forest of *Quercus* sp., 10 May 2009, GK3799 (ACAM 2010-128), coll. Ch. Chrysopoulos and K. Giatra. Trikala Prefecture: Koziakas Mt., in soil under mixed forest of *Quercus* sp. and *P.nigra*, 2 April 2013, GK6538 (ACAM 2013-073), coll. K. Papadimitriou (GenBank: MK113978); Koziakas Mt., in soil under mixed forest of *Quercus* sp. and *P.nigra*, 2 April 2013, GK6537 (ACAM 2013-074), coll. K. Papadimitriou. Ioannina Prefecture: Metsovo, in soil under pure stand of *Q.coccifera*, 18 April 2016, GK9408 (ACAM 2016-001), coll. A. Bideris (GenBank: MK113977); Metsovo, in soil under pure stand of *Q.coccifera*, 19 April 2016, GK9409 (ACAM 2016-002), coll. A. Bideris; Metsovo, in soil under pure stand of *Q.coccifera*, 19 April 2016, GK9410 (ACAM 2016-003), coll. A. Bideris; Demati, in soil under pure stand of *Q.coccifera*, 22 March 2017, GK10231 (ACAM 2017-033), coll. A. Bideris. Attica Prefecture: Katsimidi, in soil under mixed forest of *Q.ilex* and *P.halepensis*, 22 March 2016, VK4482 (ACAM 2016-004), coll. V. Kaounas (GenBank: MK113980); Katsimidi, in soil under mixed forest of *Q.ilex* and *P.halepensis*, 12 April 2016, VK4506 (ACAM 2016-005), coll. V. Kaounas (GenBank: MK113980). Ioannina Prefecture: Neochoropoulo, in soil under a mixed stand of *Q.coccifera* and *Q.ilex*, 27 April 2016, LT1183 (ACAM 2016-006), coll. V. Nakkas (GenBank: MK113976). BULGARIA. Varna, Dolishte village, in soil under pure stand of *Carpinus* sp., 07 June 2017, MSL 1945 F8517 (SOMF 29978; ACAM 2017-034), coll. R. Radev (GenBank: MK113981). Sliven, in soil under a mixed stand of *Quercusrobur*, *Corylus* sp., *Carpinus* sp. and *Acer* sp., 09 August 2017, MSL 1961 F0388 (SOMF 29979; ACAM 2017-035), coll. K. Pilasheva & P. Neikov (GenBank: MK113982).

##### Phylogenetic aspects.

**Figure 5.**
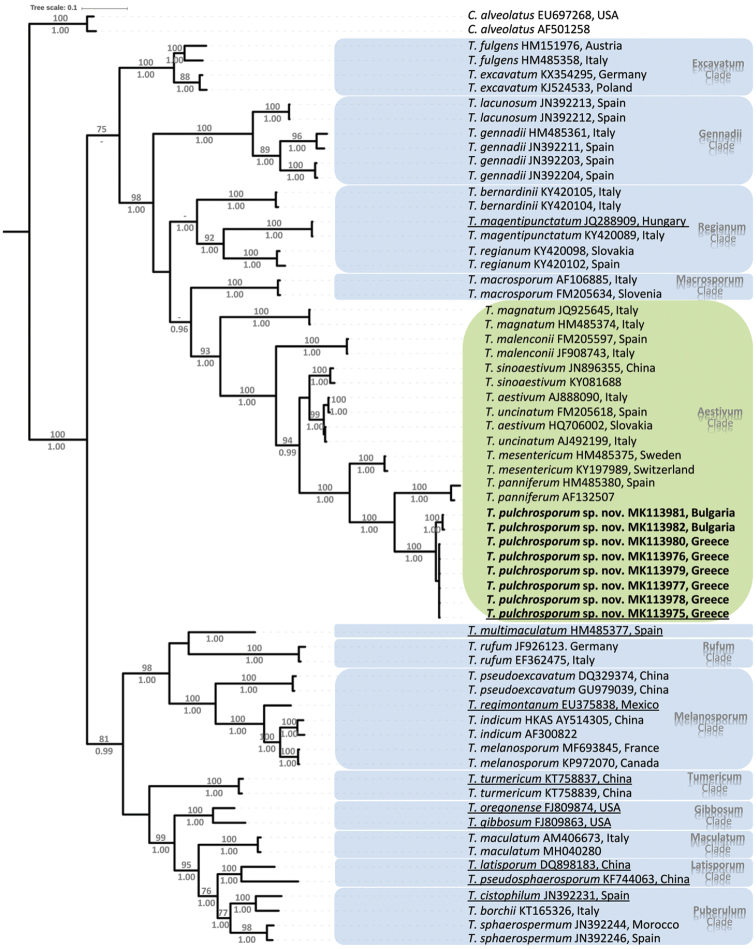
Phylogenetic tree inferred from Bayesian analysis including 62 ITS sequences assigned to 31 *Tuber* taxa, including members of major clades of the genus. Sequences are labelled with Latin binomials, GenBank accession numbers and geographic origin. *T.pulchrosporum* sp. nov. is indicated in boldface. Reference sequences deriving from type material are underlined. *Choiromycesalveolatus* (Tuberaceae) was used as the outgroup. Bootstrap (BS) values from Maximum Likelihood (ML) analysis (≥ 70%) and Posterior Probabilities (PPs) from Bayesian Inference (≥ 0.95) are shown at the nodes of branches.

The resultant ITS sequence data comprises of 64 sequences which were aligned at 780 sites, 738 of which represent the ITS1-5.8S-ITS2 region, i.e. between the end of the SSU motif (CATTA) and the beginning of LSU motif (TAGGG) (Bonito et al. 2010a). ML and BI analyses yielded similar tree topologies and only the tree inferred from the Bayesian analysis is presented (Fig. 5). The morphologically variable genus *Tuber* is monophyletic (BS: 100%, PP: 1.00) and several lineages are revealed; for the purposes of this study, the following highly supported clades were included: Aestivum, Excavatum, Gennadii, Gibbosum, Latisporum, Maculatum, Macrosporum, Melanosporum, Puberulum, Regianum, Rufum, Tumericum (=Japonicum).

According to the phylogenetic analysis performed, *T.pulchrosporum* belongs to the Aestivum clade. All eight sequences of this new taxon form a distinct highly supported subclade (BS: 100%, PP: 1.00). Greek specimens possessed almost identical ITS sequences (99.8 – 100%) and so did Bulgarian samples, whereas the comparison between collections from the two countries resulted in sequence identity values of 98.13 ± 0.08%. In total, intraspecific sequence identity values for *T.pulchrosporum* exceeded 98% (i.e. 98.05 – 100%). The new species is sister to *T.panniferum* (BS: 100%, PP: 1.00); the respective sequences demonstrated low sequence identity (73.21 – 75.08%) further evidencing their distinct taxonomic status.

## Discussion

The molecular analysis evidenced that the eight sequences representing *T.pulchrosporum* are grouped within the Aestivum clade by forming a distinct terminal group supported with high BS and PP values. The closest phylogenetic relative of *T.pulchrosporum* is *T.panniferum* Tul. & C. Tul., i.e. a Mediterranean species with analogous ecological preferences (Jeandroz et al. 2008). *T.panniferum* also exhibits a rather similar macromorphology characterised by a brownish pubescent peridium, absence of pyramidal warts and ascomata often bearing a cavity, although the tomentum is much more prominent, exhibiting thus a felted appearance. However, the microscopic features of the two species are clearly different. In *T.panniferum*, the ornamentation consists of isolated spines never exceeding 3 μm in height, while the peridial surface is covered by rusty brown hyphae which form a dense cottony mass (Montecchi and Sarasini 2000; Riousset et al. 2001; Moreno-Arroyo et al. 2005).

By morphology alone, *T.pulchrosporum* is easily distinguishable within the Aestivum clade since no other species produces ascospores bearing such a uniquely crested ornamentation. The more distant *T.aestivum* (Wulfen) Spreng. (including *T.uncinatum* Chatin) and *T.sinoaestivum* J.P. Zhang & P.G. Liu could be distinguished macroscopically thanks to their blackish peridial surface with prominent pyramidal warts and ascospores bearing a complete reticulum. Ascospores of *T.mesentericum* Vittad. show some affinity in their outline to those of *T.pulchrosporum* but they clearly possess a much more reticulate network; moreover, the peridial surface is black with pyramidal warts as in *T.aestivum*.

Although phylogenetically more distant, some other species with asci containing 1–8 ascospores may superficially resemble *T.pulchrosporum*. Hence, *T.regianum* Montecchi & Lazzari, the recently described *T.magentipunctatum* Z. Merényi, I. Nagy, Stielow & Bratek and *T.bernardinii* Gori, all belonging to the Regianum clade (Zambonelli et al. 2016; Crous et al. 2017), possess a reddish-brown to brown peridial surface with dense and rather flat warts as in the case of *T.pulchrosporum*. However, they all produce ascospores with pointed spines which are connected to form a complete reticulum. Ascomata of *T.malenconii* Donadini, Riousset, G. Riousset & G. Chev and *T.pseudoexcavatum* Y. Wang, G. Moreno, Riousset, Manjón & G. Riousset also show a macroscopic resemblance to *T.pulchrosporum*, with their rough indistinctly warty peridial surface (black for the former and brown for the latter), often with a similar basal cavity as well. However, ascospores of both *T.malenconii* and *T.pseudoexcavatum* have short spines, basally/broadly connected, exhibiting a more or less regular reticulum (Donadini et al. 1979; Manjón et al. 2009). Therefore, the unique type of ornamentation of *T.pulchrosporum* ascospores clearly distinguishes it from all species with similar macroscopic appearance.

## Supplementary Material

XML Treatment for Tuber pulchrosporum
